# K.Vita: a feasibility study of a blend of medium chain triglycerides to manage drug-resistant epilepsy

**DOI:** 10.1093/braincomms/fcab160

**Published:** 2021-07-23

**Authors:** Natasha E Schoeler, Michael Orford, Umesh Vivekananda, Zoe Simpson, Baheerathi Van de Bor, Hannah Smith, Simona Balestrini, Tricia Rutherford, Erika Brennan, James McKenna, Bridget Lambert, Tom Barker, Richard Jackson, Robin S B Williams, Sanjay M Sisodiya, Simon Eaton, Simon J R Heales, J Helen Cross, Matthew C Walker, K.Vita Study Group, Sanjeev Rajakulendran, Aikaterini Vezyroglou, Suresh Pujar, Judith Kalser, Christin Eltze, Sophia Varadkar, Robert Robinson, Shamima Rahman

**Affiliations:** 1 UCL Great Ormond Street Institute of Child Health, London, UK; 2 Great Ormond Street Hospital for Children, London, UK; 3 National Hospital for Neurology and Neurosurgery, London, UK; 4 UCL Queen Square Institute of Neurology, London, UK; 5 Chalfont Centre for Epilepsy, Chalfont-St-Peter, UK; 6 Vitaflo (International) Ltd, Liverpool, UK; 7 University of Liverpool, Liverpool, UK; 8 Department of Biological Sciences, Royal Holloway University of London, Surrey, UK

**Keywords:** ketogenic, decanoic acid, C10, seizure

## Abstract

This prospective open-label feasibility study aimed to evaluate acceptability, tolerability and compliance with dietary intervention with K.Vita, a medical food containing a unique ratio of decanoic acid to octanoic acid, in individuals with drug-resistant epilepsy. Adults and children aged 3–18 years with drug-resistant epilepsy took K.Vita daily whilst limiting high-refined sugar food and beverages. K.Vita was introduced incrementally with the aim of achieving ≤35% energy requirements for children or 240 ml for adults. Primary outcome measures were assessed by study completion, participant diary, acceptability questionnaire and K.Vita intake. Reduction in seizures or paroxysmal events was a secondary outcome. 23/35 (66%) children and 18/26 (69%) adults completed the study; completion rates were higher when K.Vita was introduced more gradually. Gastrointestinal disturbances were the primary reason for discontinuation, but symptoms were similar to those reported from ketogenic diets and incidence decreased over time. At least three-quarters of participants/caregivers reported favourably on sensory attributes of K.Vita, such as taste, texture and appearance, and ease of use. Adults achieved a median intake of 240 ml K.Vita, and children 120 ml (19% daily energy). Three children and one adult had ß-hydroxybutyrate >1 mmol/l. There was 50% (95% CI 39–61%) reduction in mean frequency of seizures/events. Reduction in seizures or paroxysmal events correlated significantly with blood concentrations of medium chain fatty acids (C10 and C8) but not ß-hydroxybutyrate. K.Vita was well accepted and tolerated. Side effects were mild and resolved with dietetic support. Individuals who completed the study complied with K.Vita and additional dietary modifications. Dietary intervention had a beneficial effect on frequency of seizures or paroxysmal events, despite absent or very low levels of ketosis. We suggest that K.Vita may be valuable to those with drug-resistant epilepsy, particularly those who cannot tolerate or do not have access to ketogenic diets, and may allow for more liberal dietary intake compared to ketogenic diets, with mechanisms of action perhaps unrelated to ketosis. Further studies of effectiveness of K.Vita are warranted.

## Teaser

See Auvin (https://doi.org/10.1093/braincomms/fcab234) for a scientific commentary on this article.

Schoeler et al. report that K.Vita, a decanoic acid-rich medical food, was well-tolerated when introduced gradually and flexibly to individuals with epilepsy. Seizures or paroxysmal events reduced by 50% (95% CI 39–61%). Only 4/61 (7%) patients had ß-hydroxybutyrate levels >1 mmol/l. K.Vita may be valuable for individuals with drug-resistant epilepsy.

## Graphical Abstract

**Graphical Abstract:**
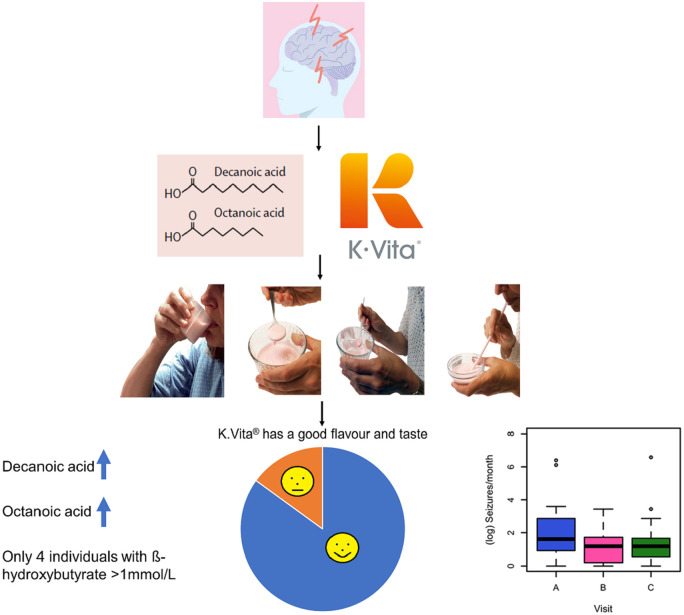

## Introduction

Ketogenic diets (KDs) are high-fat, low-carbohydrate diets, and are the management option of choice for certain neurometabolic disorders and an effective treatment option for individuals with drug-resistant epilepsy.^1^ Despite their efficacy, the stringent dietary restriction of KDs greatly impacts patients and their carers. Medium chain triglycerides (MCTs) enable decreased fat and increased protein and carbohydrate intake compared to long chain triglyceride-based KDs, although MCTs may be unacceptable due to their oily taste and distinctive mouth and throat feel.^2^ MCT products can be flavoured or incorporated into recipes to enhance palatability, but this may be inconvenient and time-consuming.^3^^,^^4^

The canonical view of the mechanism of action of KDs is through ketone production,^5^ despite the lack of correlation of ketone levels with seizure reduction.^6^^,^^7^ This has driven increasing interest in the role of MCT-derived fatty acids, in particular decanoic acid (C10).^8^ C10 is able to cross the blood–brain barrier and decreases excitatory neurotransmission and network excitability *in vitro*,^9^ increases seizure threshold in mice,^10^ and increases mitochondrial number plus respiratory chain and catalase activity.^11^^,^^12^ These effects may be potentiated by C8, which spares C10 from oxidation in neuronal-like cells.^13^

This potentially beneficial effect of C10 on energy metabolism and network excitability, in combination with improved palatability of MCT, may offer a simplified approach to dietary management of epilepsy. K.Vita contains a higher proportion of C10 to C8 (80:20) than currently available medical foods (generally 40:60 C10:C8). This trial aimed to evaluate compliance, acceptability, tolerability and biochemical impact of K.Vita in the dietary management of individuals with drug-resistant epilepsy.

## Materials and methods

### Ethics

Favourable ethical opinion for this single-arm, prospective feasibility study was granted by London—City & East Research Ethics Committee (15/LO/1979). Written informed consent was obtained from all participants (or their carers) in the study. The study was registered with Clinicaltrials.gov (NCT02825745).

### Study product

K.Vita is a ready-to-use, palatable thickened flavoured liquid containing 80:20 C10:C8 triglyceride, in 120 ml packets.

### Sponsor

This study was funded by Vitaflo (International) Ltd. The sponsor created, produced and distributed K.Vita and were involved in the concept and study design, analysis of data and reviewing the manuscript. They were not involved in data collection, the writing of the initial manuscript or the decision to submit the manuscript for publication.

### Participants

A target sample size of 40 children and 40 adults was determined on the basis of obtaining sufficient information to perform reliable sample size estimates for future later phase studies. Participants were adults, aged >18, and children aged 3–18 years (under 3 s were excluded owing to Foods for Special Medical Purposes regulations) with epileptic seizures, or associated paroxysmal non-epileptic events [for individuals with glucose transporter type 1 deficiency syndrome (GLUT1-DS) or Alternating Hemiplegia of Childhood (AHC)], despite adequate levels of antiseizure medicines (ASMs). Children had a clinical or genetic diagnosis of Dravet syndrome or another early-onset epilepsy resulting from a confirmed or presumed genetic mutation. Inclusion criteria were absence of medical conditions that contra-indicated use of MCT, for example, medium-chain acyl-coenzyme A dehydrogenase deficiency; participant and/or parent/guardian able to comply with the study protocol; written, informed consent. Exclusion criteria were children <3 years old; freedom from seizures or paroxysmal events for >4 weeks at the time of recruitment; currently following a KD; females who were pregnant or planning to become pregnant during the study.

### Setting

Potential participants were identified by their consultants during routine epilepsy clinics at Great Ormond Street Hospital for Children (GOSH, London, UK), The National Hospital for Neurology and Neurosurgery (London, UK) and The Chalfont Centre for Epilepsy (Chalfont-St-Peter) from June 2016 to March 2018.

### Schedule of events

The study consisted of three visits (A, B and C): baseline, 5 and 12 weeks after starting K.Vita, plus regular telephone or email contact. At visit A, the target amount of K.Vita was determined, dependent on participant age. For children, this was a maximum of 17.5% daily energy requirements by visit B and 35% daily energy requirements, or two packets, by visit C. The target amount for adults was a maximum of one packet daily by visit B and two packets daily by visit C. K.Vita was introduced incrementally, according to gastrointestinal (GI) tolerance. A protocol amendment (May 2017) allowed greater flexibility in the speed of introducing K.Vita (no maximum amount of time was set, whereas prior to the amendment introduction was generally over one week) and amount taken (clarification that the maximum daily amounts were not a target to be reached). The amount was divided into three or four equal servings, taken at regular intervals daily, as part of a meal, snack or enteral feed. When taken orally, K.Vita was consumed unadulterated, or mixed into cordial or food such as yogurt. When taken via feeding tube, it was administered either immediately before or after, or mixed into, an enteral feed, with a water flush directly after administration.

Participants and/or parent/guardians were given guidance on excluding high-refined sugar foods and beverages, such as sweetened drinks, fruit juice, confectionary and breakfast cereals, to balance the additional calories from K.Vita, if appropriate, and to optimize the nutritional quality of the participant’s diet. Starchy carbohydrates, such as bread, pasta, rice and potatoes, were not restricted. If tube fed, participants kept their usual enteral feed/regime, with adjustments to balance the calories from K.Vita if necessary. If there was concern about the nutritional adequacy of a participant’s diet during the study, dietary advice was given and/or a daily micronutrient supplement was started. K.Vita tolerance was reviewed and daily intake adjusted, as appropriate. A daily record was kept of K.Vita intake, GI symptoms and frequency of seizures or paroxysmal events. No changes were made to concurrent medications, unless advised by the investigator for severe seizure escalations.

At each visit, anthropometric measures, 3-day diet history and seizure review were undertaken. At visit C, participants and/or parent/guardians completed an acceptability questionnaire relating to their experience of using K.Vita. The option was given to continue K.Vita; participants then received their usual care, as provided pre-study.

### Biochemistry

Biochemical blood and urine analysis was performed at all visits to assess patient suitability and monitor clinical safety. Samples were analysed at GOSH or UCL Great Ormond Street Institute of Child Health. Plasma levels of free medium chain fatty acids and ß-hydroxybutyrate (BHB) were measured at all visits by negative ion chemical ionization gas chromatography mass spectrometry or positive ion chemical ionization gas chromatography mass spectrometry, respectively.

### Statistical analysis

The main outcomes of the feasibility study were tolerability, acceptability and compliance with K.Vita, assessed via the metrics of: number of patients to withdraw from the study, number of adverse events reported throughout the study, participant comments on appearance, taste, flavour preference, texture, ease of consumption, ease of use, method of use, packaging and presentation of K.Vita, number of patients to discontinue or reduce K.Vita intake, total quantity of K.Vita consumed, and proportion of daily calories from K.Vita consumed compared to starch, sugar, protein and fat intake from dietary intake. Continuous data were described as mean or median [interquartile range (IQR)] and categorical data were presented as frequencies of counts with associated percentages.

Data on seizure/paroxysmal event frequency in the first, second and third 4-week blocks of the study were modelled using repeated measures random effects modelling, adjusting for data source (study diary or physician-documented count) and patient age (child versus adult) as fixed effects, and participant identifier as a random intercept. Regression modelling included baseline seizure/event rate as an adjusting covariate with seizure/event counts modelled on the log + c scale. The rate of seizures was modelled on the log + 0.5 scale following inspection of the model residuals. Sensitivity analyses were performed discounting patients who changed dose or number of ASMs taken during the intervention period and to account for changes to the study protocol.

Plasma C8, C10 and BHB levels were compared using Mann–Whitney test. Correlation between biochemistry parameters was assessed using Spearman’s rank correlation. Associations between biomarker data and seizure/event rates were explored by including the biomarker expression data as a fixed effect in the random effects model. Plasma C10 and seizure/event frequency were transformed using a log(x + c) transformation. Plasma C8 was not included in the model owing to multicollinearity. All analyses were conducted using *R: A Language and Environment for Statistical Computing (Version 3)*.^14^

### Data availability

The data that support the findings of this study are available from the corresponding author, upon reasonable request.

## Results

**Figure 1:**
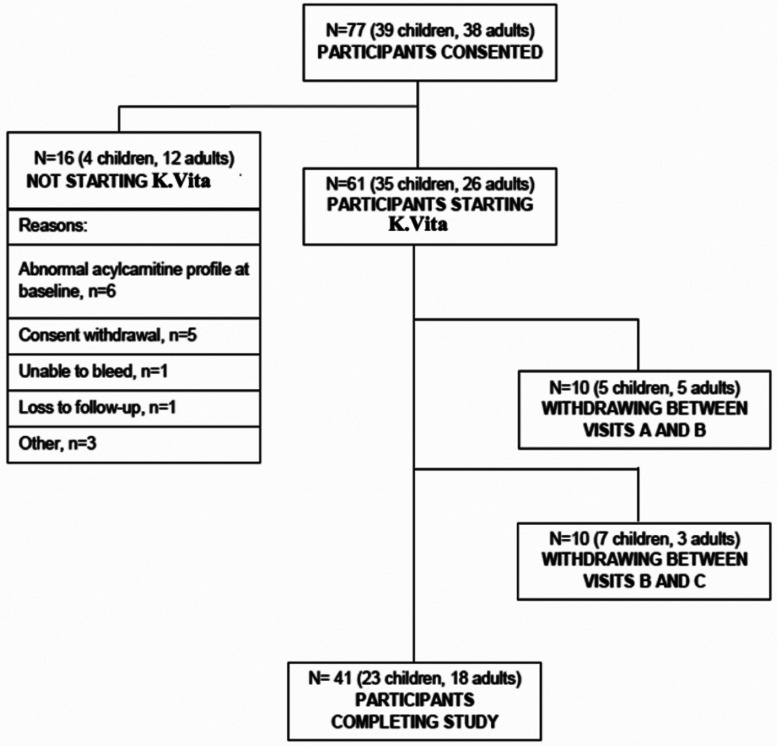
Flowchart of participants.

Seventy-seven individuals were consented, of whom 61 (35 children, 26 adults; 59% female) started K.Vita (Fig. 1; Table 1). The children had refractory genetic epilepsies, AHC or GLUT1-DS, and the adults had mainly focal epilepsies (Table 1), all of whom had failed multiple ASMs: median 3 ASMs (IQR: 4–7) for children and median 10 ASMs (IQR: 8–15) for adults, who completed the trial.

**Table 1:** Demographic data and baseline characteristics of participants who started K.Vita

		Participants starting K.Vita (total *n* = 61)
Sex (self-reported), *n* (%)	Female	36 (59%)
	Male	25 (41%)
Age (years)	Mean	21.1
	Median	15.7
	Min, Max	2.8^a^, 68
Age categories (years), *n* (%)	3–18	35 (56%)
	≥18	26 (44%)
Ethnicity, *n* (%)	Caucasian	51 (84%)
	Asian or Asian British	3 (5%)
	Mixed Race	3 (5%)
	Black or Black British	3 (5%)
	Other	1 (2%)
Epilepsy syndrome/diagnosis—children	Dravet syndrome	8 (23%)
	Genetic epilepsy (Other)^b^	13 (37%)
	Presumed genetic epilepsy	6 (17%)
	Alternating Hemiplegia of Childhood (AHC)	5 (14%)
	GLUT1 deficiency syndrome	3 (9%)
Epilepsy syndrome/diagnosis—adults		
	Focal epilepsy	15 (58%)
	Genetic generalized epilepsy	6 (23%)
	GLUT1 deficiency syndrome	1 (4%)
	Unknown	4 (15%)

a One child started prior to third birthday due to current clinical state, at the discretion of the investigator.

b Includes mutations in PCHD19 (*n* = 2), SCN1A not associated with Dravet Syndrome (*n* = 2), CHD2, CDKL5, ALG13, SCN8A, KCNA2 and FBXL4, tetrasomy 18p10, translocation between chromosomes 3 and 9, and Ch9q22 deletion.

Of the 61 participants who started K.Vita, 20 (33%, 19 children and 1 adult) had previously been on a KD. Of these 20, 9 (45%) had stopped the KD for personal or social reasons, predominantly due to patient and/or caregiver non-compliance, 7 (35%) due to lack of efficacy, 2 (10%) as they had been on the diet for >2 years, and 2 (10%) due to unknown reasons. Of those who started K.Vita, 7/35 (20%) children and 4/26 (15%) adults had been previously referred for KD treatment but did not start owing to clinical concerns, social circumstances or caregiver apprehension about what the KD entailed. Of 26 adults, 21 (81%) had no access to KD services.

Sixty-six per cent (23/35) of children and 69% (18/26) of adults completed the trial (Fig. 1), giving an overall withdrawal rate of 20/61 (33%). The most common reasons for withdrawal were GI side effects—predominantly abdominal pain/discomfort, diarrhoea and/or vomiting—reported by 5/12 (42%) children and 6/8 (75%) adults, who discontinued. Other reasons for discontinuation (some participants reported multiple reasons) were behaviour change (*n* = 2 children), reduced appetite (*n* = 1 child), possible exacerbation of seizures (*n* = 1 child and *n* = 1 adult), sore throat (*n* = 2 adults), lack of clinical effectiveness (*n* = 1 child and *n* = 1 adult) and unknown/lost to follow-up (*n* = 4 children and *n* = 1 adult).

Exploratory analyses of the impact of a protocol amendment, which altered the speed of introduction of K.Vita, showed that the number of participants withdrawing from the study due to adverse events reduced from 32% (14/38) under the initial protocol to 18% (3/17) in the revised protocol. The proportion of participants who started K.Vita but then withdrew from the study due to any reason reduced from 36% (16/38) under the initial protocol to 24% (4/17) in the revised protocol.

Ninety-one per cent (21/23) of children and 56% (10/18) of adults who completed the study, chose to remain on K.Vita post-study. Those who declined to remain on K.Vita did so due to perceived lack of clinical effectiveness.

### Tolerability

The most frequently reported GI symptoms were abdominal bloating/feeling full, excessive flatulence and constipation (Table 2). GI symptoms peaked during the introduction period, then tended to decrease. There was a peak in instances of diarrhoea in weeks 11 and 12, primarily due to two participants (seemingly unconnected to K.Vita for one participant and unknown for the other). Non-GI reported adverse side effects, considered to be possibly, probably or highly probably related to K.Vita were infrequent (25 reports in total) and most (72%) were mild in severity (Table 3).

**Table 2:** Number of reports of gastrointestinal symptoms in study cohort

	**Symptom reports in cohort, *n* = 39** ^a^					
	Weeks 1–4		Weeks 5–8		Weeks 9–12	
	Median (IQR)	Highest number of reports per person	Median (IQR)	Highest number of reports per person	Median (IQR)	Highest number of reports per person
Vomiting	0 (0–0)	6	0 (0–0)	2	0 (0–0)	6
Nausea	0 (0–1)	8	0 (0–0)	12	0 (0–0)	10
Abdominal bloating/feeling full	0 (0–2)	28	0 (0–0)	28	0 (0–0)	25
Abdominal pain/discomfort	1 (0–6)	18	0 (0–1)	18	0 (0–0)	15
Excessive burping	0 (0–0)	13	0 (0–0)	5	0 (0–0)	1
Excessive flatulence	0 (0–2)	24	0 (0–0)	28	0 (0–0)	21
Diarrhoea	0 (0–1)	10	0 (0–0)	14	0 (0–1)	14
Constipation	0 (0–0)	20	0 (0–0)	28	0 (0–0)	21

a Number of participants who returned study diaries.

**Table 3:** Number of reports and severity of non-gastrointestinal adverse side effects during the study period

Adverse side effect	Number of reports classified as ‘Mild’	Number of reports classified as ‘Moderate’	Number of reports classified as ‘Severe’	Total number of reports
Mood swings/behavioural change	2	1	–	3
Sore throat	3	–	–	3
Decreased appetite	1	1	–	2
Fatigue	1	1	–	2
Amenorrhea	1	–	–	1
Bed wetting	1	–	–	1
Increase in biting episodes	–	1	–	1
Cramps	–	1	–	1
Dizziness	1	–	–	1
Food phobia	–	1	–	1
Headache	–	–	1	1
Heartburn	1	–	–	1
Hypoglycaemic event	1	–	–	1
Itchy skin	1	–	–	1
Pain on urination	1	–	–	1
Pale stools	1	–	–	1
Possible seizure exacerbation	1	–	–	1
Reflux	1	–	–	1
Small red patches on face on consumption of unflavoured version of K.Vita	1	–	–	1

Prior to the protocol amendment, participants reported a greater degree of nausea, abdominal bloating and abdominal pain than those who entered into the study following the amendment. Conversely, those who entered under the amended recommendations showed greater amounts of excessive flatulence and constipation.

### Acceptability

Of 15 caregivers and 19 adults who returned the acceptability questionnaire, 84% agreed/strongly agreed that K.Vita had good flavour and taste, 88% liked the appearance and colour of K.Vita, 77% liked the texture, mouthfeel and consistency of K.Vita, and 88% agreed/strongly agreed that it was easy to take K.Vita and to follow advice about reducing sugar intake. One-third of adults felt that K.Vita was helpful for their epilepsy, compared to two-thirds of caregivers.

### Compliance

Eighty-seven per cent (41/47) of participants complied with the recommended amount of K.Vita at visit B [median (IQR) = 84.5 ml (60–100) or 15% total energy for children, and 120 ml (120–120) for adults]; 28/38 (74%) complied at visit C [median (IQR) = 120 ml (90–180) or 19% total energy for children and 240 ml (240–240) for adults].

In the 22/23 (96%) children for whom data are available for visits B and C, the median (IQR) intake of K.Vita was 90.5 ml (63.75, 100) at visit B and 120 ml (90, 180) at visit C. Overall, 18/22 (82%) increased intake between the two visits, 2/22 (9%) had no change in intake and 2/22 (9%) reduced their intake. In the 16/18 (89%) adults for whom data are available for visits B and C, a median (IQR) intake of 120 ml (120, 120) was observed at visit B and 240 ml (230, 240) at visit C. 14/18 (89%) increased intake between the two visits, 1/18 (6%) showed no change and 1/18 (6%) reduced intake. A total of 6/40 (15%) participants reduced K.Vita intake or discontinued prior to visit C.

### Assessment of daily energy and macronutrient intakes

Four children were partially or totally enterally fed and were successfully given K.Vita via their feeding tubes. Mean daily energy intake was 1452 kcal (range 532–2941 kcal, *n* = 34) for children and 1446 kcal (range 676–2263 kcal, *n* = 26) for adults at visit A; 1788 kcal (range 788–4097 kcal, *n* = 29) for children and 1981 kcal (range 1419–3277 kcal, *n* = 19) for adults at visit B; 2009 kcal (range 992–3367 kcal, *n* = 22) for children and 2167 kcal (range 1113–3276 kcal, *n* = 26) for adults at visit C.

**Figure 2:**
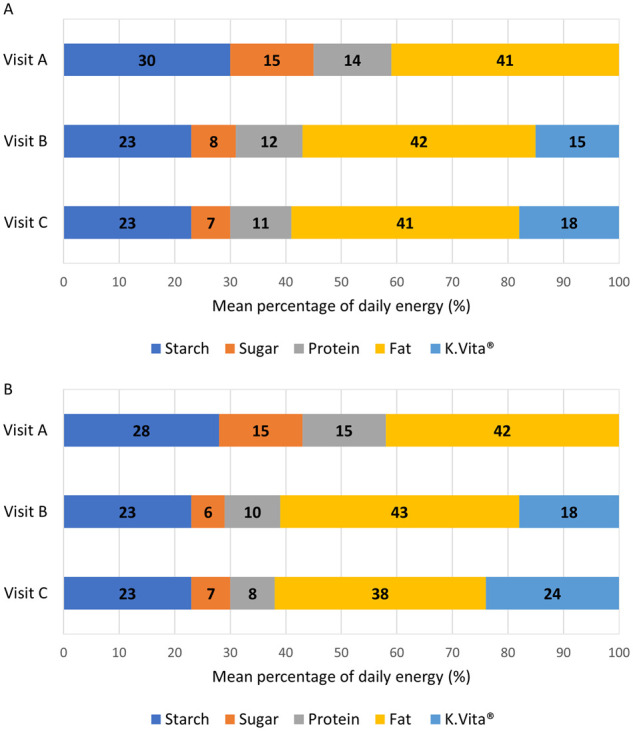
**Percentage energy from macronutrients and K.Vita at study visits. Mean percentage of daily energy from macronutrients** (visit A, B and C) and K.Vita (visits B and C) (A in children; B in adults)

Mean energy from dietary fat remained stable from visits A to C: 41% daily energy at both visits in children and 42% to 38% daily energy in adults; K.Vita provided an additional mean 15% daily energy at visit B and 18% at visit C in children, and 18% daily energy at visit B and 24% at visit C in adults (Fig. 2). In accordance with study guidelines, intake of sugar decreased following visit A in children and adults (Fig. 2).

The mean proportion of energy from protein decreased from visits A to C (Fig. 2), although actual mean daily intakes for children remained stable (mean 52.9 g at visit A; 53.9 g at visit C). For adults, mean daily intakes reduced by 1.8 g (mean 51.4 g at visit A; 49.6 g at visit C).

Eighty-six per cent (18/21) of children met their daily Average Requirement (AR), and/or Population Reference Intake for protein throughout the study. Sixty-nine per cent (11/16) of adults met their daily AR for protein (0.66 g/kg/day), which was no different to baseline.

### Anthropometry

There was no significant change in body weight (mean change +1.57 kg, range −3.0 to +10.8 kg in children; −0.41 kg, range −4.6 to +4.7 kg in adults) or body mass index (BMI) (*z*-scores −0.21, range −1.5 to +0.45 for children; mean change +0.1 kg/m^2^, range −2.2 to +3.3 kg/m^2^ for adults). Weight loss of 4.6 kg was observed in one adult with a baseline BMI 33.1 kg/m^2^; weight gain of 4.7 kg was observed in another adult with a baseline BMI 18.2 kg/m^2^. In both cases, weight change was desired and clinically appropriate. One child with GLUT1-DS gained 10.8 kg (+14 percentiles, UK-World Health Organization growth charts), due to greater range and quantity of foods available, following recent discontinuation of the KD. Reported energy intake confirmed this and dietetic guidance was provided.

### Biochemistry

**Figure 3:**
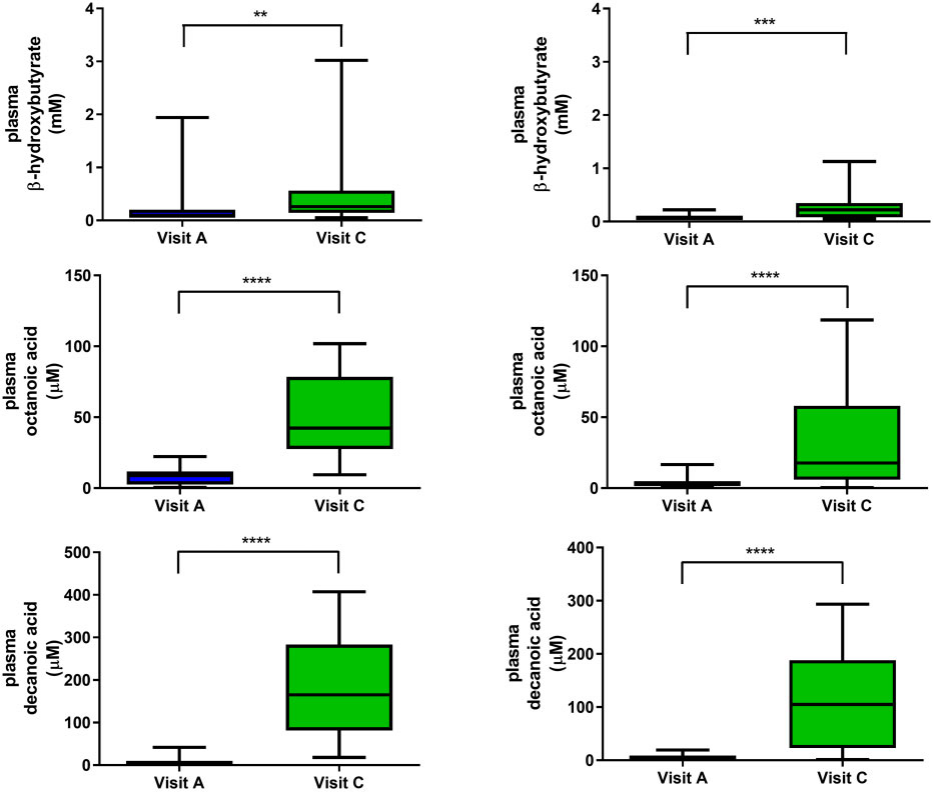
**Beta-hydroxybutyrate and medium chain fatty acid levels at baseline and visit C. Plasma beta-hydroxybutyrate and medium chain fatty acid levels measured in participants at both baseline and at visit C: median, interquartile range and range** (Left panels in children; Right panels in adults). **P < 0.01, ***P < 0.001, ****P < 0.0001 (Mann–Whitney test).

There were no significant changes in routine biochemical parameters from baseline to visit C (Table 4), nor any changes that were deemed clinically significant, independent of whether participants completed the trial or not. Most participants exhibited a very mild yet significant increase in plasma BHB throughout the trial [median (range) 0.07 (0.05–0.22) mmol/l at visit A and 0.22 (0.05–1.13) mmol/l at visit C in adults; 0.095 (0.05–1.94) mmol/l at visit A and 0.26 (0.05–3.02) mmol/l at visit C in children, Fig. 3]. Only three children (17%) and one adult (7%) became ‘ketotic’ (BHB > 1 mmol/l); 5 children (28%) and 2 adults (15%) had BHB >0.5 mmol/l. C8 and C10 fatty acid levels increased significantly from baseline to visit C, with adults showing a less pronounced increase [median (range) 17.7 (0.3–118.6) μmol/l C8 and 104.8 (1.1–293.5) μmol/l C10 at visit C] than children [median (range) 42.3 (9.4–101.8) μmol/l C8 and 165.1 (18.3–407.4) μmol/l C10 at visit C; Fig. 3]. The relative contribution of C8 and C10 to the free fatty acid pool increased from 3.2 ± 2.8% at baseline to 37.7 ± 14.2% at visit C. At visit C, C10 accounted for mean 77.0 ± 10.0% and C8 mean 23.0 ± 10% of the total medium chain fatty acids in blood, consistent with the 80:20 C10: C8 ratio in K.Vita.

**Table 4:** Biochemical parameters measured at baseline and visit C (study completers only)

	Children		Adults	
	**Visit A** **Mean (range)**	**Visit C** **Mean (range)**	**Visit A** **Mean (range)**	**Visit C** **Mean (range)**
Glucose (mmol/l)	4.3 (2.8–5.7)	4.3 (3.1–6.4)	4.5 (3.6–5.8)	4.1 (3.3–5.1)
Triglycerides (mmol/l)	1.18 (0.53–3.80)	1.35 (0.54–3.91)	1.3 (0.46–3.83)	1.3 (0.57–3.91)
NEFA (mmol/l)	0.55 (0.17–1.03)	0.62 (0.20–1.19)	0.4 (0.08–1.03)	0.5 (0.23–0.99)
Cholesterol (mmol/l)	4.1 (3.0–5.2)	4.2 (0.7–5.3)	5.2 (3.9–7.6)	5.4 (3.7–7.5)
Urea (mmol/l)	4.8 (2.6–7.0)	4.3 (1.9–7.3)	4.8 (2.8–7.6)	4.3 (3.2–5.6)
Creatinine (μmol/l)	49 (23–86)	46 (22–69)	74.3 (59–93)	72.6 (54–88)
Sodium (mmol/l)	142 (138–148)	142 (138–146)	142 (135–146)	143 (131–147)
Potassium (mmol/l)	4.4 (3.5–7.0)	4.5 (3.4–7.1)	4.1 (3.5–>7.0)	4.0 (3.5–6.7)
ALT (U/l)	22 (9–32)	25 (14–36)	30.4 (8–64)	47.6 (8–284)
ALP (U/l)	157 (61–266)	150 (85–271)	77.3 (40–124)	71.9 (42–106)
Bilirubin (µmol/l)	8 (0–13)	7 (4–12)	10.1 (4–15)	9.5 (6–16)
Albumin (g/l)	44 (37–51)	44 (39–50)	45.4 (36–51)	45.8 (40–54)
Urate (µmol/l)	249 (98–405)	275 (139–420)	274.3 (102–488)	307.8 (146–523)
Total CO_2_ (mmol/l)	23 (18–29)	23 (16–27)	28 (20–33)	26.8 (22–34)
Calcium (mmol/l)	2.35 (2.15–2.53)	2.36 (2.16–2.58)	2.4 (2.2–2.69)	2.4 (2.24–2.61)
Magnesium (mmol/l)	0.90 (0.81–1.03)	0.89 (0.78–1.01)	0.90 (0.74–0.98)	0.8 (0.76–0.91)
Phosphate (mmol/l)	1.49 (1.02–1.86)	1.42 (0.97–1.82)	1.2 (0.97–1.82)	1.2 (0.92–1.47)

### Clinical efficacy

Thirty-nine per cent (9/23) of children and 56% (10/18) of adults who completed the study returned the study diaries with quantifiable records of seizures or (for one individual with GLUT1-DS and one individual with AHC) paroxysmal non-epileptic events. Physician-documented seizure/event counts were reviewed for all others; four children had insufficient data to allow for quantification of seizures.

Thirty-eight per cent (6/16) (38%) of children and 50% (8/16) of adults achieved ≥50% seizure or event reduction. Some syndromes seemed to respond particularly well: both children with AHC, who completed the trial, had ≥50% reduction in seizures/paroxysmal events; one child with GLUT1-DS and gait difficulties, but without current seizures, became free of paroxysmal events.

**Figure 4:**
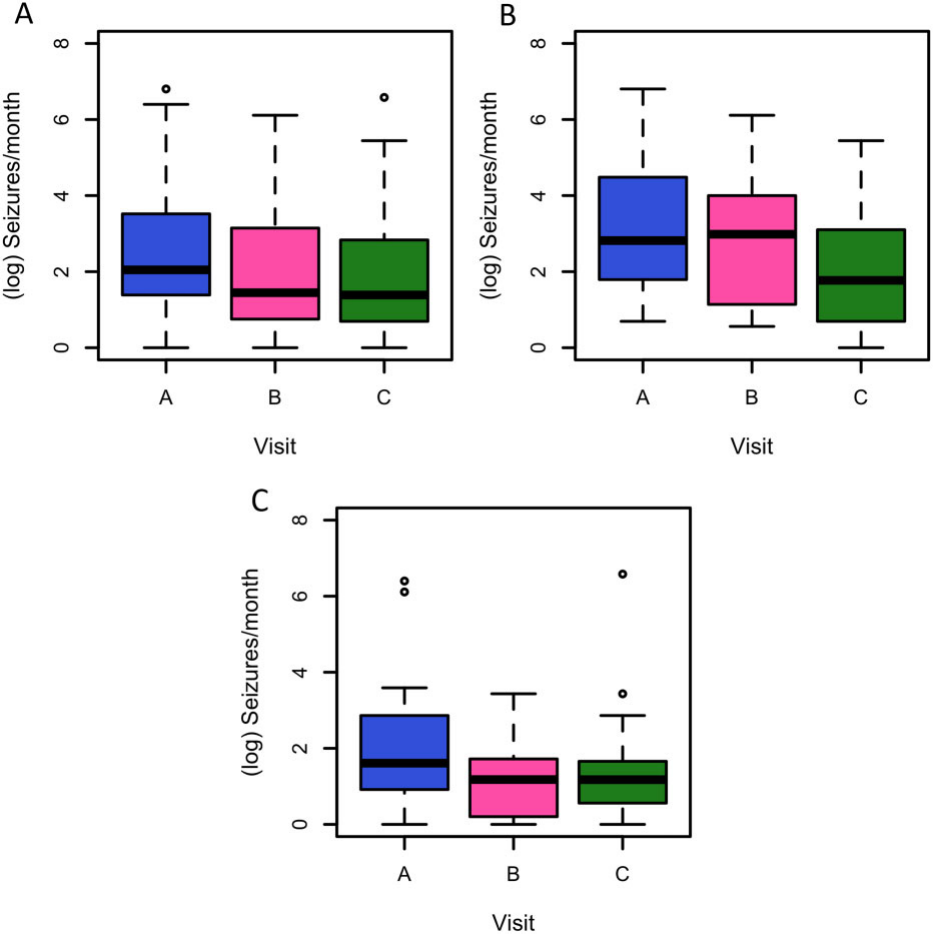
**Seizures or paroxysmal events at visits A, B and C. Number of reported seizures or paroxysmal events at visits A, B and C, in 4-week epochs** (**A** in all participants; **B** in children; **C** in adults). Analysis is based on *n* = 113 observations from 44 individuals, using linear mixed modelling, adjusting for data source (study diary or physician-documented count) and patient age (adult versus child) as fixed effects, and participant identifier as a random intercept. The reduction between baseline and visit B has a *t*-value of −3.255 (*P* = 0.0015) and the reduction between baseline and visit C has a *t*-value of −4.959 (*P* < 0.0001) (all participants).

The estimated mean rate of seizures or paroxysmal events in children and adults was 14.31 at visit A (weeks 1–3), 9.12 at visit B and 7.22 at visit C: overall there was a 50% (95% CI 39–61%) reduction in seizures/events between visits A and C. There was a statistically significant reduction in estimated number of seizures/events from visits A–B (*Z* = −3.399, *P* = 0.001) and visits A–C (*Z* = −5.122, *P* < 0.001), adjusting for the source of data (diary versus physician documentation), patient age and study visit (Fig. 4).

None of the sensitivity analyses, excluding participants with seizure data who changed dose or number of ASMs within the intervention period (*n* = 7), and comparing participants who started prior to (*n* = 43) or after the protocol amendment (*n* = 13), had any substantive impact on the interpretation of the study results (Supplementary Table 1).

### Association of clinical response with biochemical parameters

Plasma C10 concentration negatively correlated with seizure/event frequency (*P* < 0.0001). Plasma C8 and C10 were highly correlated (Pearson’s *R*^2^ 0.87), and plasma BHB was weakly correlated with C8 (*R*^2^ 0.19), C10 (*R*^2^ 0.21) and C8:C10 ratio (*R*^2^ 0.01). There was no significant association between plasma BHB concentrations and seizure/event frequency (χ^2^ = 0.0793, *P* = 0.778).

## Discussion

This open-label trial showed that K.Vita, when introduced gradually in accordance to individual tolerance, was favourably accepted in our cohort alongside minimal dietary modification. Adverse side effects were predominantly mild and resolved with dietetic support. Adults who completed the study were, on average, able to comply with the daily target amount; most children better tolerated lower amounts. Although the study was uncontrolled and not designed to determine clinical response, mean frequency of seizures/paroxysmal events was significantly reduced, despite low levels or absence of ketosis and the fact that seizure frequency was only documented from the time of introduction.

The target amount of MCT from K.Vita was based on the minimum percentage of daily energy requirements known to be efficacious in the MCT KD.^4^ GI symptoms experienced in this study are commonly associated with MCT^15^ and were no different to those reported in individuals following KDs.^16^ GI symptoms from K.Vita decreased over time and were mitigated by phased introduction, which is not always the case for KDs. For example, 33–45% children following KDs suffer constipation during the first 3 months, and these high rates continue thereafter, irrespective of whether a classical or MCT KD is followed^17^; in our study, reported incidences of constipation declined by the end of 12 weeks. Other adverse effects were infrequent, mostly non-specific and likely unrelated to K.Vita. Attrition rates due to adverse effects in our study were low after greater flexibility was allowed with the time-scale for K.Vita introduction and quantity taken. At all stages, scheduled contact with the dietitian to provide support, advice and encouragement, helped reduce the incidence of GI side effects, and prevented early discontinuation. In randomized controlled studies of children and adults on KDs,^18^ diet discontinuation rates prior to 3 months are as high as 55%.

The majority of participants or caregivers in our study reported favourably on the sensory attributes of K.Vita and ease of use in conjunction with minimal dietary restriction. In contrast, reported grievances from individuals who have commenced a KD include issues with food preparation and restricted choices;^19^ approximately half of adults and 20% of children have discontinued dietary treatment, sometimes despite clinical effectiveness, owing to the intense effort required for food selection and preparation, social restrictions or refusal to comply with the diet.^20^^,^^21^ The restraints of conventional KDs may deter people from starting dietary treatment, with only 14–30% of successfully screened adult patients enrolling in dietary trials.^20^ This is reflected in our cohort who started K.Vita, of whom almost half who had previously been on a KD had discontinued it due to personal or social reasons, including non-compliance, and almost 20% were offered KD but chose not to start. Such issues are likely to be overcome with the use of K.Vita in this specific population.

Adults and children reduced their intake of high-sugar foods and beverages, in line with study dietary advice. Total carbohydrate intakes remained higher than those typically prescribed as part of an MCT KD.^22^ Percentage energy from protein decreased, although actual intakes compared to requirements were adequate for most participants and mean daily intake was maintained for children. Reported energy intake increased for most participants, although this did not generally result in unwarranted increases in weight/BMI. Participants’ energy requirements may have been lower than calculated due to immobility from neurological impairment and reduced activity levels associated with epilepsy.^23^^,^^24^ Assessment of dietary intakes by the dietitian, although highly trained and experienced in this technique, was reliant on self-reporting, which likely impacted the determination of accurate energy intakes at all time points.^25^ The need for dietetic input with this intervention should be highlighted, albeit substantially less compared to that needed for KDs.^26^

Although this uncontrolled study was not designed to determine clinical response, and the proportion of completed study diaries was low, the mean reduction of seizures/events observed in our cohort compares favourably to KD efficacy,^18^ particularly given the super-refractory nature of the population studied, and the fact that seizure documentation was only started as K.Vita was initiated (there was no pre-study baseline recorded). The correlation of seizure/event reduction with plasma concentrations of fatty acids also supports an effect of the diet. Efficacy was observed in a range of seizure types and epilepsy syndromes, similar to those that respond to KDs. Noteworthy improvement was observed in individuals with AHC and in the one patient with GLUT1-DS and associated gait difficulties, who had not had achieved such clinical improvement with previous KD treatment. These findings need to be evaluated further, with consideration of alternative methods of daily recording of seizures/events.

With the exception of isolated cases, participants were not in a state of continued ketosis, typically classified as BHB >1 mmol/l (although definitions range from as low as 0.2 or 0.5 mmol/l),^27^^,^^28^ and BHB levels detected were markedly lower than those seen in individuals following a KD. This reduces the risk of hyperketosis and eliminates the often onerous task of daily ketone testing. C8 and C10 plasma levels at visit C were in close agreement with levels observed in patients on the MCT KD.^29^^,^^30^ Similar levels were seen in mouse brains following C10-enriched feeding^10^^,^^31^ and in *in vitro* studies with the optimum concentration for mitochondrial proliferation in neuronal-like and fibroblast cell cultures, approximately 250 μM.^11^^,^^12^

The suggested beneficial clinical effect of dietary intervention using K.Vita with low levels or absence of ketosis, supports the putative role of C8 and C10 in enhancing brain-energy metabolism and subsequent reduction of seizure activity, probably independent of ketosis. *In vitro* studies have shown that C10, but not C8, increased cellular mitochondrial number in neuronal-like and fibroblast cell cultures, and enhanced mitochondrial respiratory chain activity.^11^^,^^12^ C10 alone exhibited antioxidant properties by elevating catalase activity, providing evidence of an additional role of C10 in neuroprotection against oxidative stress. Moreover, C10 but not C8 has been shown to decrease excitatory transmission and to reduce network excitability *in vitro*.^9^ In neurons, C10 is oxidized at about 20% of the rate of C8, and in the presence of C8, C10 oxidation is even further decreased,^13^ suggesting that C8 exerts a sparing effect on C10 catabolism, minimizing its degradation and contributing to the maintenance of brain levels. This is supported by previous work showing beneficial clinical effects of combined octanoic and decanoic acid compared to decanoic acid alone.^10^ The positive correlation of C10 with seizure/event response in our study is consistent with these hypotheses but, due to the clear relationship between C8, C10 and BHB, we cannot rule out that other metabolites also play a role.

Consumption of K.Vita, alongside additional dietary changes, was generally well accepted, tolerated and complied with in our cohort, as long as a flexible approach was taken to introduction and target intake. This study was designed to determine tolerability of a nutritional product rather than primarily to determine effect on seizures or paroxysmal events. Nevertheless, in this group of complex, highly drug-resistant children and adults, we observed a significant reduction in frequency of seizures, or paroxysmal events for some participants, noting that the study was not blinded, nor placebo-controlled. Our findings show promising development for liberalizing dietary management for epilepsy, and clinical resource savings. K.Vita addresses an unmet need for those who cannot access KD services. Further study is warranted; however, in particular, for individuals for whom observance of a KD may prove too challenging and outweigh any clinical benefits, and for specific disorders dependent on a KD, such as GLUT1-DS, and conditions with paroxysmal neurological events, such as AHC, with use of K.Vita alongside minimal dietary modification compared to a traditional KD, as in this study.

## Supplementary material

Supplementary material is available at *Brain Communications* online.

## Supplementary Material

fcab160_Supplementary_Data

## Abbreviations

AHC =: alternating hemiplegia of childhood
AR =: average requirement
ASMs =: antiseizure medicines
BHB =: ß-hydroxybutyrate
BMI =: body mass index
GI =: gastrointestinal
GLUT1-DS =: glucose transporter type 1 deficiency syndrome
GOSH =: Great Ormond Street Hospital for Children
IQR =: interquartile range
KDs =: ketogenic diets
MCTs =: medium chain triglycerides
