# Supplementary material for: K.Vita: a feasibility study of a blend of medium chain triglycerides to manage drug-resistant epilepsy
Source: Brain Commun. 2021 Jul 23;3(4):fcab160. doi: 10.1093/braincomms/fcab160 (PMC8557697; doi:10.1093/braincomms/fcab160)
Supplement: fcab160_Supplementary_Data [file fcab160_supplementary_data.pdf]

Supplementary Table 1: Sensitivity analyses to assess the impact of changing dose or number of anti-seizure drugs during the intervention period, and to account for changes to the study protocol, on number of reported seizures or paroxysmal events at visits A, B and C

	Final Model		Sensitivity Analysis 1		Sensitivity Analysis 2		Sensitivity Analysis 3	
	est (se)	Pval	est (se)	Pval	est (se)	Pval	est (se)	Pval
(Intercept)	2.66 (0.266)	<0.001	2.72 (0.272)	<0.001	2.88 (0.305)	<0.001	1.97 (0.553)	<0.001
sourceDum	-0.61 (0.524)	0.121	-0.49 (0.531)	0.179	-1.11 (0.594)	0.031	1.06 (0.993)	0.143
childDum	-0.34 (0.523)	0.261	-0.35 (0.531)	0.254	-0.52 (0.595)	0.193	-0.74 (1.101)	0.251
visitB	-0.45 (0.132)	<0.001	-0.46 (0.136)	<0.001	-0.46 (0.148)	0.001	-0.42 (0.315)	0.089
visitC	-0.68 (0.134)	<0.001	-0.7 (0.139)	<0.001	-0.68 (0.155)	<0.001	-0.68 (0.28)	0.008

Sensitivity analysis 1: Excluding patients with any change in dose or number of anti-seizure drugs taken during the intervention period

Sensitivity analysis 2: Only including participants who entered the study prior to the protocol amendment (implemented 1st June 2017)

Sensitivity analysis 2: Only including participants who entered the study after the protocol amendment (implemented 1st June 2017)

Supplementary Information: K.Vita® Study Group

Dr Sanjeev Rajakulendran, National Hospital for Neurology and Neurosurgery, London
Dr Aikaterini Vezyroglou, Great Ormond Street Hospital for Children, London
Dr Suresh Pujar, Great Ormond Street Hospital for Children, London
Dr Judith Kalser, Great Ormond Street Hospital for Children, London
Dr Christin Eltze, Great Ormond Street Hospital for Children, London
Dr Sophia Varadkar, Great Ormond Street Hospital for Children, London
Dr Robert Robinson, Great Ormond Street Hospital for Children, London
Professor Shamima Rahman, Great Ormond Street Hospital for Children, London
